# Supplementary material for: Translation of Experimental Findings from Animal to Human Biology: Identification of Neuronal Mineralocorticoid and Glucocorticoid Receptors in a Sectioned Main Nerve Trunk of the Leg
Source: Cells. 2023 Jul 5;12(13):1785. doi: 10.3390/cells12131785 (PMC10340435; doi:10.3390/cells12131785)
Supplement: Supplementary file 1 [file cells-12-01785-s001.zip › cells-2374480-supplementary.pdf]

cells

MDPI

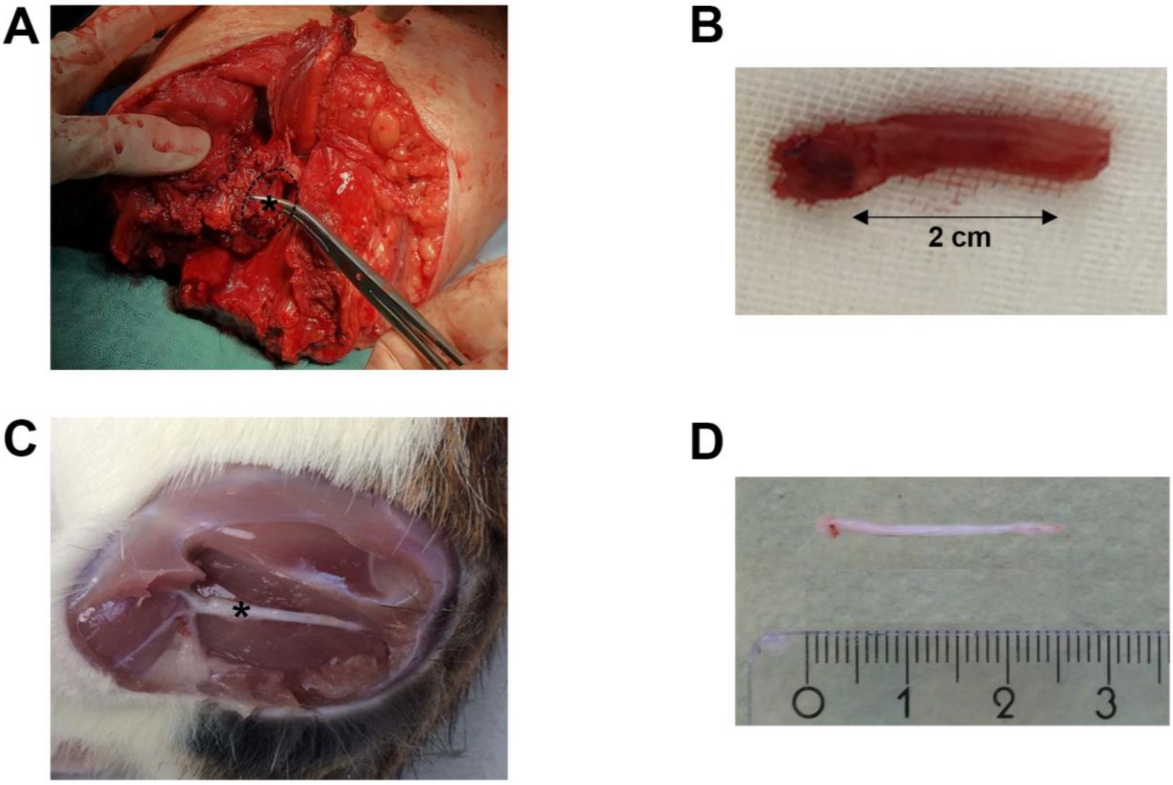

Figure S1. Peripheral nerve trunk preparation. (A) Excision of a human nerve trunk (B) during amputation. The *marker in the photographs shows the position of the nerve. (C) Preparation of the rat sciatic nerve before its branching. (B, D) show the relative dimensions of the nerve trunk in humans compared to rats.

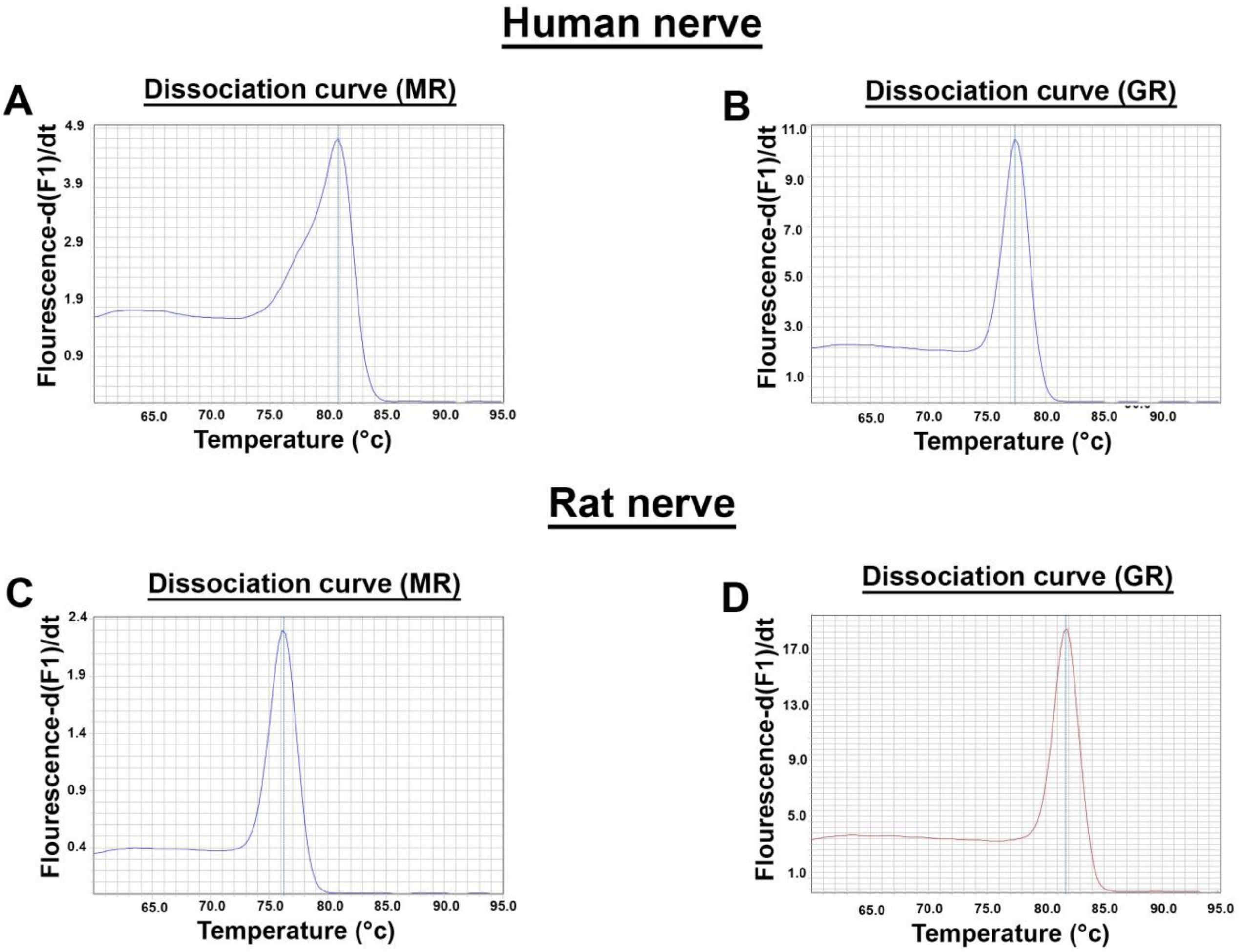

Figure S2. Graphical representation of the temperature-dependent dissociation curves of the target specific primer pairs from the template DNA. Each individual dissociation curve represents the primer specific fluorescence change over time with the identification of a single melting temperature at which half of the primer is dissociated from the specific target DNA (MR = mineralocorticoid receptor, GR = glucocorticoid receptor.

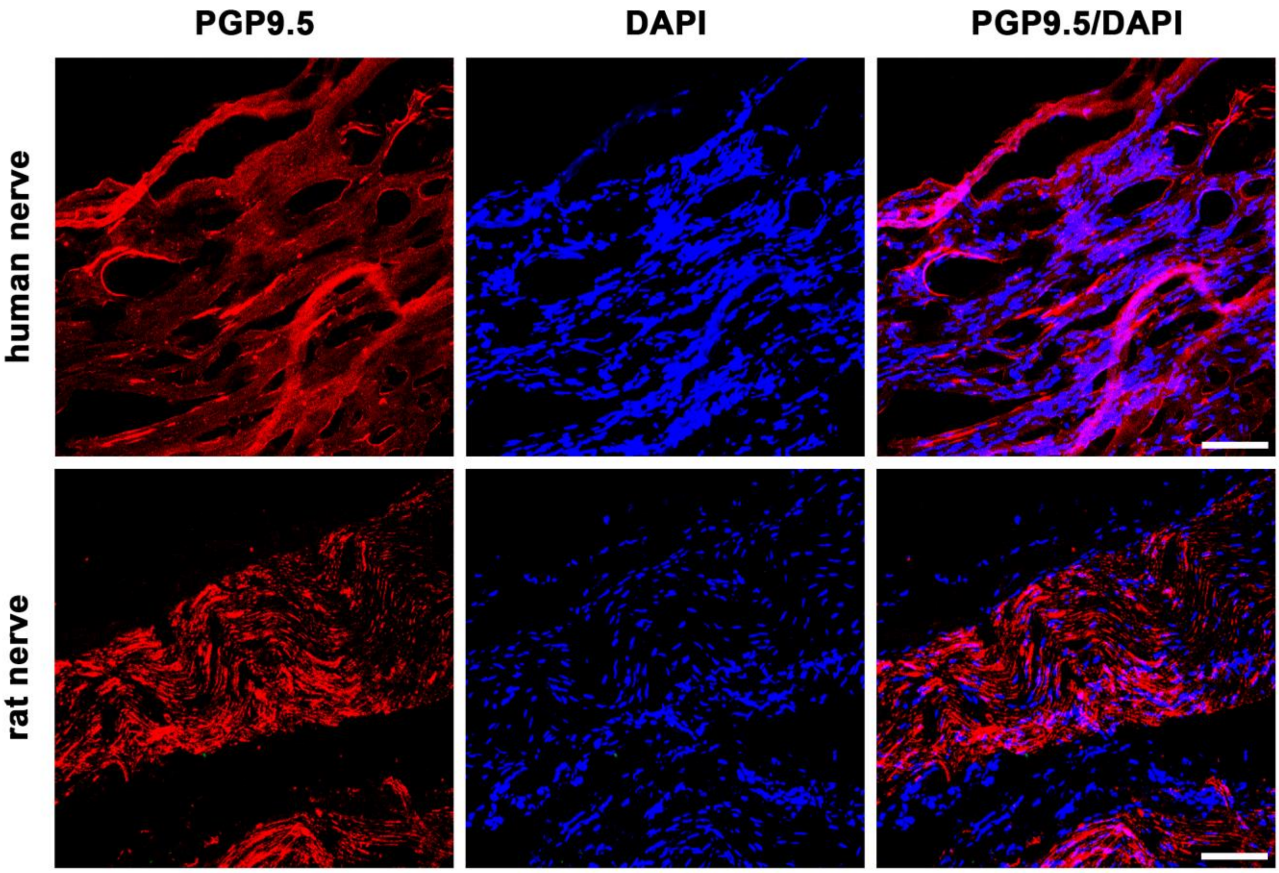

Figure S3. Confocal microscopy of peripheral nerve sections from human (upper row) and rat nerve trunk (lower row). Tissue sections were immunostained with the pan-neuronal polyclonal rabbit antibody anti-PGP9.5 (red fluorescence) together with DAPI (blue-fluorescent DNA stain) for the identification of nuclear structures of cells in peripheral nerve sections of human (upper row) compared to rat (lower row). The pictures confirm that the chosen tissue sections represent peripheral nerve tissue without any overt pathology. Bar = 20 μm.

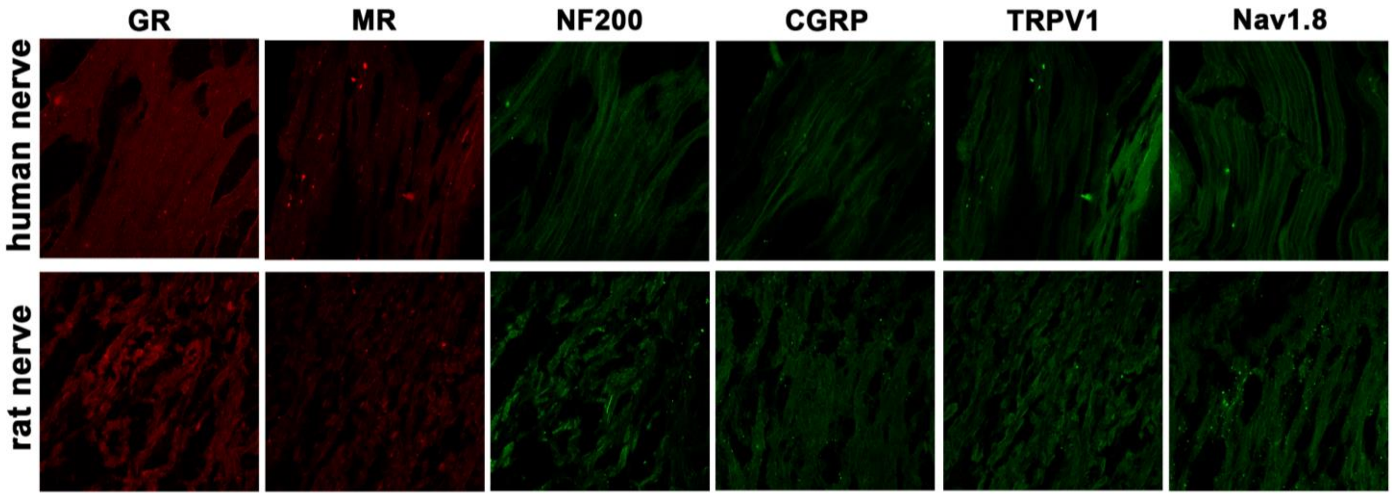

Figure S4. Immunohistochemical negative control experiments. The results of our negative control immunohistochemical experiments in which we omitted each individual primary antibody (GR, MR, NF200, CGRP, TRPV1, Nav1.8) and incubated only with the secondary antibody show that auto-fluorescence is low confirming the specificity of primary antibody staining in Figures 2–6.

Table S1. Characterization of primary antibodies used.

Antigen	Immunogen	Manufacturer, Species, Type, Catalogue Number, Reference	Dilution used
GR	A part of the rat GR transcription modulation domain	A gift from M. Kawata (Kyoto Prefectural University of Medicine, Japan), rabbit polyclonal, [45]	1:3.000
MR	A peptide mapping at rMR 1–18	A gift from Gomez-Sanchez (Mississippi Medical Center, USA) mouse monoclonal, [15]	1:100
CGRP	Synthetic entire calcitonin gene-related peptide	Peninsula Laboratories (CA, USA), guinea pig polyclonal, # T-5027, [47]	1:1.000
NF200	Carboxy terminal tail segment of dephosphorylated NF200	Sigma-Aldrich (USA), mouse monoclonal # N0142/N52, [46]	1:1.000
TRPV1	VR1 C-terminus (TRPV1)	Neuromics MN, USA), guinea pig polyclonal # GP14100 # [44]	1:1.000
Nav1.8	A peptide (C)EDEVAAKEGNSPGPQ corresponding to residues 1943-1957 of rat Nav1.8	Sigma-Aldrich (USA), polyclonal rabbit, # S2071, [3]	1:1.000

Table S2. List of Primers for Taqman RT-PCR.

Gene	Access. Nr.	Forward	Reverse
Human MR	NM_000901.4	5′-AAAGAGCAGTGGAAGGGCAA-3′	5′-TGAAGTCTGCAAGCAGGACAA-3′
Human GR	NM_0001762	5′-TGCTCCTTCTGCGT TCACAA-3′	5′-CCATCAGTGAATATCAACTCTGGC-3′
18S	M10098	5′-AAACGGCTACCACATCCAAG-3	5′-CCTCCAATGGATC CTCGTTA-3′
Rattus norvegicus MR	NM_013131.1	5′-CCAAGGTACTTCCAGGATTTAAAAAC-3′	5′-AACGATGATAGACACATC CAAGAATACT-3′
Rattus norvegicusGR	NM_012576.2	5′-CATCTTCAGAACAGCAAAATCGA-3′	5′-AGGTGCTTTGGTCTGTGGGATA-3′
S18	NR_046237	5′-CGGCTACCACATCCAAGGAA-3′	5′-GCTGGAATTACCGCGGCT-3′
